# Similarity Index–Probabilistic Confidence Estimation of SARS-CoV-2 Strain Relatedness in Localized Outbreaks

**DOI:** 10.3390/epidemiologia3020019

**Published:** 2022-05-06

**Authors:** Mahmood Y. Bilal

**Affiliations:** 1 Department of Pathology, University of Iowa Hospitals and Clinics, Iowa City, IA 52242, USA; mahmood-bilal@uiowa.edu or mahmood.bilal@va.gov; 2 Pathology and Laboratory Medicine, Iowa City VA Health Care System, 601 US-6 W, Iowa City, IA 52246, USA

**Keywords:** SARS-CoV-2, disease outbreaks, RNA-seq, molecular epidemiology, biostatistics

## Abstract

Outbreaks of SARS-CoV-2 can be attributed to expanding small-scale localized infection subclusters that eventually propagate into regional and global outspread. These infections are driven by spatial as well as temporal mutational dynamics wherein virions diverge genetically as transmission occurs. Mutational similarity or dissimilarity of viral strains, stemming from shared spatiotemporal fields, thence serves as a gauge of relatedness. In our clinical laboratory, molecular epidemiological analyses of strain association are performed qualitatively from genomic sequencing data. These methods however carry a degree of uncertainty when the samples are not qualitatively, with reasonable confidence, deemed identical or dissimilar. We propose a theoretical mathematical model for probability derivation of outbreak-sample similarity as a function of spatial dynamics, shared and different mutations, and total number of samples involved. This Similarity Index utilizes an Essen-Möller ratio of similar and dissimilar mutations between the strains in question. The indices are compared to each strain within an outbreak, and then the final Similarity Index of the outbreak group is calculated to determine quantitative confidence of group relatedness. We anticipate that this model will be useful in evaluating strain associations in SARS-CoV-2 and other viral outbreaks utilizing molecular data.

## 1. Introduction

Lineage identification in conjunction with virion mutational analysis showed rapid expansion shortly post the SARS-CoV-2 (CoV-2) pandemic that began in Wuhan/China in late 2019. This in part was due to increased accessibility of Next Generation Sequencing (NGS) technology within research and diagnostic laboratories. During that time, the utility of targeted genomic sequencing was used to address a combination of research and clinical questions related to CoV-2 infections. First, it permitted tracking of temporal viral mutagenesis. Second, defining CoV-2 mutational repertoire allowed identification of infections that could be clinically addressed with targeted treatments, including monoclonal antibodies, in critically ill patients [1,2]. Third, laboratories were equipped with molecular data for epidemiological investigations. Specifically, sequencing data would allow confirmation or rejection of the hypothesis that a certain group of infected individuals carry the same CoV-2 virion, given similar spatiotemporal parameters.

The utility of molecular sequencing data can be applied within a pandemic or to localized outbreak clusters that occur within hospitals or assisted living facilities. In the latter case, public algorithms such as NextClade (v1.14.1) can be useful, qualitatively, for defining similarity or dissimilarity in localized CoV-2 outbreak strains [3]. This method was used in our clinical laboratory as part of the Sequencing for Research Clinical and Epidemiology (SeqFORCE) (SeqFORCE) group within the Veteran Health Administration. Other studies put forth excellent probabilistic models for defining infection kinetics within the COVID-19 pandemic. To this end, Shadabfar et al. proposed an extended susceptible-exposed-infected-vaccinated-recovered (SEIVR) epidemic model [4]. The modified SEIVR model allows the quantitative prediction of the COVID-19 spreading profile in society while being careful to implement uncertainties in data collected from research and clinical laboratories. Nevertheless, current tools for assessment of localized outbreak clusters are generally in the domain of qualitative assessment. We propose that quantitative analyses for localized outbreaks should be included in order to compute a measure of confidence for CoV-2 strain relatedness. To this end, we derived a sequencing-based mathematical model that can be applied as a quantitative confidence estimator of how similar (or dissimilar) the samples are, in terms of combined Essen-Möller ratios and probabilities (Similarity Index or “SI”). The Similarity Index is purely based on empirical mutational frequencies obtained from circulating CoV-2 sequenced strains. This model was empirically tested in our laboratory in a similar manner that we demonstrate with the two clinical cases shown in this work. As such, we believe that this procedure can be utilized as part of future molecular epidemiological analyses for localized viral outbreak investigations and/or improved upon with other algorithms.

## 2. Materials and Methods

### 2.1. Sample Collection, RNA Extractions, and Next Generation Sequencing (NGS) of CoV-2 Genome

CoV-2 RNA was extracted utilizing Qiagen’s QIAamp Viral RNA kit per manufacturer’s guidelines (Qiagen, 19300 Germantown Road, Germantown, MD, USA). Exclusion criteria for any outbreak investigation included samples that had >30 cycle threshold (Ct) as defined by quantitative reverse-transcription PCR (qRT-PCR). All patient CoV-2 viral sequences in this study were used by permission of originating facilities.

Sequencing of CoV-2 genomes was performed by a clinically validated ARTIC-v3 assay. Preparations of CoV-2 NGS libraries were performed with QIAseq SARS-CoV-2 Primer Panel per manufacturer’s guidelines (Qiagen). All single libraries were loaded into Illumina’s reagent cartridge (300 cycle v2) on a standard flow cell at 7 pM (Illumina, 5200 Illumina Way, San Diego, CA, USA). Sequencing quality controls, including cluster density, total reads, and percent reads reaching Q30, were all within optimal ranges provided by Illumina. Secondary quality controls, provided by Illumina’s DRAGEN COVID Lineage software (v3.5.5) that reads and quantifies the sequencing files, were all within acceptable ranges. Attained values for median depth and breadth of coverage were at least 500× and 95%, respectively.

### 2.2. Bioinformatics and Consensus Sequences of CoV-2 Variants

FASTQ files were obtained from the Illumina MiSeq and then uploaded onto Illumina’s *DRAGEN COVID Lineage* software (v3.5.5). The consensus FASTA files were then uploaded to NextClade’s algorithm (v1.14.1) in order to define each strain’s mutational repertoire. NextClade can be found at (https://clades.nextstrain.org/).

### 2.3. Calculation of Nucleotide Mutation Frequency

COVID CG was utilized for attaining the frequency of mutational occurrence during the date range of 1 April 2021–14 December 2021 (COVIDCG, v2.5.1-beta1, https://covidcg.org/). This query included 1127754 CoV-2 sequences where the Delta CoV-2 variant was dominant [5,6]. Note that the time-framed frequencies reported here may slightly change for the aforementioned period, since data from COVID CG are continuously updated with new sequences.

Each mutation frequency was derived from the equation:

(1) $$\mathrm{Frequency}=\frac{\mathrm{Total}\;\mathrm{Occurrence}\;\mathrm{of}\;\mathrm{Mutation}}{\mathrm{Total}\;\mathrm{Number}\;\mathrm{of}\;\mathrm{CoV}-2\;\mathrm{Genomes}\;}$$

### 2.4. Geometrical Representations

Graphs presented in this manuscript were obtained from Desmos software, API v1.7.0 (https://www.desmos.com/).

## 3. Similarity Index—A Theoretical Model

Frequency estimates of specific mutations appearing within CoV-2 genomes can be produced by utilizing the number of times a certain mutation is observed divided by total genomes analyzed (Equation (1)). The probability (*P*) multiplication rule can then be employed in this scenario to infer the *P* of a certain mutation appearing in multiple CoV-2 genomes selected at random [7]. From our experience with molecular epidemiology, we observed that the CoV-2 ORF1a region contains a degree of nucleotide instability (i.e., more informative) when compared to the Spike region. This is true within the CoV-2 Delta strain, and similar observations were noted within Omicron’s ORF1a regions. Regions with relative genomic instabilities can be used to compare outbreak strains qualitatively in their mutational repertoire utilizing low frequency mutations as a first-tier choice. Here, were propose rather the quantitative utility of mutational frequencies incorporating the full mutational repertoire within ORF1a (i.e., both low and high frequency mutations). It is important to note that, due to temporal mutational dynamics, we should expect a degree of undulation within each nucleotide site [8,9]. For this reason, frequency calculations should be dependent on recent circulating variants, not based on the bulk of total CoV-2 viral sequences with strains that are no longer circulating. For example, applying frequency data from the Delta wave to samples collected during the Omicron wave can be substantially misleading. Date ranges of observed mutations can be set within COVIDCG online software.

We hence propose the use of the ORF1a genomic region to produce a combined, time-framed, frequency profile of the mutational repertoire integrated with the number of samples containing this profile (i.e., *P* product-rule). These calculations are then applied to the Essen-Möller equation initially produced to prove or disprove paternity [10]. The merged equations are utilized to produce a Similarity Index as we show in the following derivations.

### 3.1. Similarity Index–Derivation of Base Equation

Mutational profiles, factored in as mutational frequencies, can be applied when comparing two or more strains that are part of a localized outbreak. Here, two variables are of most importance. The first is each nucleotide’s mutational frequency. The second is the number of outbreak samples carrying that same mutation.

Therefore, the *P* (*P_m_*) of a certain mutation with Frequency (*F*) to appear together in a pool of CoV-2 samples at random or by chance is:

$$P_{m}=F_{s1\;}\times F_{s2}\times F_{s3}\times F_{sn}$$

where *F* relates to the calculated mutational frequency (Equation (1)), and (*s*1, *s*2, *s*3, *sn*) relate to the individual outbreak samples carrying the exact mutation with frequency *F*. Since all samples are carrying the same mutation with the same *F*, the equation can be simplified to the following form:

$$P_{m}=F^{sn}$$

where *sn* denotes the total number of samples carrying a mutation with frequency *F*. Next, in an outbreak scenario, we are rather interested in defining the *P* that a certain mutation will not appear together at random (*P_mn_*). Thus, the above equation can be transformed to the following form:

(2) $$P_{mn}=1-F^{sn}$$

where *F* relates to a single mutational frequency, and *sn* relates to the total sample number where all samples are carrying the same mutation with frequency *F*.

**Figure 1:**
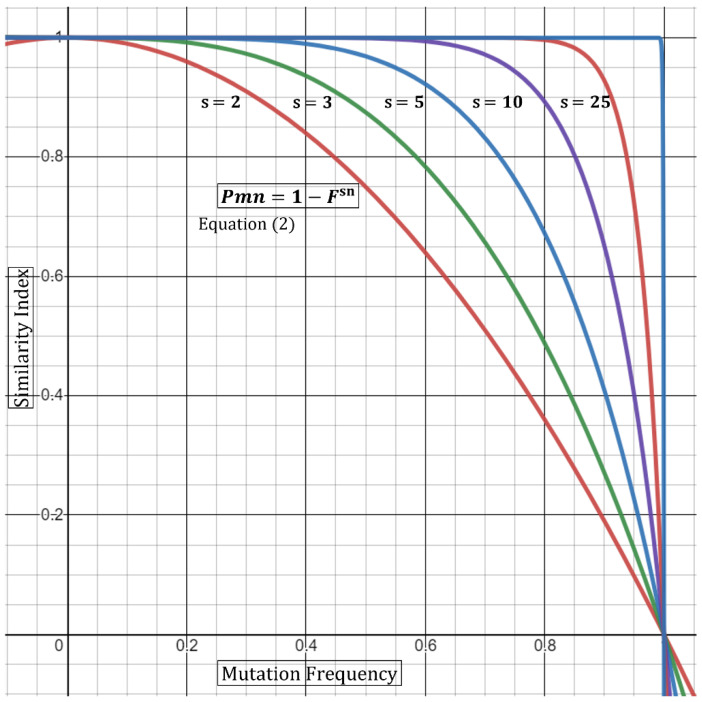
Graphical representation of Equation (2). The graph demonstrates that the probability of a certain mutation (*Pmn*) not to occur randomly is dependent on both the frequency of the mutation (*F*) and the number of samples carrying such mutation (*sn*)—Here, *sn* is varied, and *F* is kept constant. An increase in *sn* produces a larger *Pm*.

Thus, Equation (2) states that the *P* of a certain mutation not appearing at random is dependent on the number of samples as well as the mutational frequency carried by *sn* samples. We can also extrapolate from this base formula that incorporating a large outbreak sample number, *sn*, will compensate for a higher frequency (e.g., 0.15–0.65). In other words, five outbreak samples carrying a mutation with 0.3 (30%) frequency produce a higher *P* in samples that are related, compared to only two samples (Table 1). Alternatively, a low or rare mutational frequency would also raise the *P* in a smaller outbreak sample (Table 1). Geometrical representation of this argument is displayed in Figure 1. As the frequency (*F*) of shared mutations approaches zero, the *P* of association approaches 1 (100%). Similarly, as the number of samples, *sn*, carrying a mutation with multiplied-frequency (*F*) approaches infinity, the *P* of association drifts towards one. One limitation in the utility of mutation frequencies in *P* calculations is that mutations should be independent. Specifically, utilizing mutations that are co-dependent will produce misleading outputs utilizing the *P* multiplication rule [7]. To this end, Fang et al. utilized a concurrence-ratio whereby two single nucleotide variants can be assessed for the likelihood of coexisting in the same viral genomes [11].

### 3.2. Incorporation of Mutational Differences

Heterogeneity of the mutational repertoire in outbreak samples is evidence towards non-similarity, depending on the degree of divergence. Therefore, it would be misleading to base the *P* model (Equation (2)) entirely on identical/unique mutations. We specifically observed in our epidemiological investigations that although strains within an outbreak can carry unique mutations, some nevertheless had non-identical mutational signatures. It would be important to estimate quantitatively the relatedness of these strains based on both similar and dissimilar mutations. The main question is, how confident are we that the strains on-hand are related? The answer to this can in part be effectuated by incorporating dissimilar mutational frequencies within the final *P* value in order to normalize for mutational differences between samples.

To this end, we propose the use of a modified Essen-Möller’s *W* value as a base-equation:

$$\left(W=\frac{X}{X+Y}\right)$$

In its original form, *W* combines two hypotheses: X (paternity) and Y (non-paternity). Essen-Möller proposed this in order to include both possibilities wherein X + Y becomes a probability of 1 [10].

In our case, we seek to calculate *W* by summing two *P*s (i.e., similar or dissimilar strains based on mutational profiles) and then dividing the *P* of interest (similar) by the sum, thence attaining an index on which we shall name here the Similarity Index (SI) [10]. To this end, Equation (2) and Essen-Möller’s equation are coalesced as follows for comparing multiple (≥2) samples:

$$SI=1-\frac{F1^{s1}+F2^{s2}+Fn^{sn}\;}{\left(F1^{s1}+F2^{s2}+Fn^{sn}\right)+\left(F1_{d}^{s1}+F2_{d}^{s2}+Fn_{d}^{sn}\right)}$$

where *F*1→*Fn* and *F_d_*1→*F_d_n* relate to the shared and non-shared (d = different) mutations with a frequency of *F*, respectively. *s*1→*sn* relates to the total sample number associated with the frequency *F*1→*Fn*. For example, if *F*1 is (0.05), and *s*1 relates to 5 samples, then *F*1 ^s^^1^ = 0.05^5^. This would be followed by calculations and summing of all frequencies (*F*2 *^s^*^2^, *F*3 *^s^*^3^, *Fn^sn^*) relating to each specific CoV-2 nucleotide mutation. In essence, (*F*1 *^s^*^1^ + *F*2 *^s^*^2^ + *Fn^sn^*) present in both the numerator and denominator would be equivalent to “X” in Essen-Möller’s ratio. In contrast, (*F*1 *d^s^*^1^+ *F*2 *d^s^*^2^+ *Fnd^sn^*) is equivalent to “Y” in in Essen-Möller’s ratio.

This equation allows a multi-sample approach incorporating the differences in mutations, frequencies, and sample number which all contribute to the Similarity Index. The convolution of multi-sample comparison is evident when comparing samples carrying a host of identical mutations along with mutations only present within a certain subset of the group. It therefore becomes important to add in the factor of *s*, where it can guide the statistical “swaying” power of each mutation present as shown geometrically in Figure 1.

Based on this, the final equations can be summarized as follows:

(3) $$SI=1-\frac{\sum_{i=1\;}^{n}F^{si}i}{\sum_{i=1}^{n}F^{si}\;i+\sum_{i=1}^{n}F_{d\;}^{si}\;i}$$

where the product summation relates that every frequency of a shared mutation (*F*) is summed until all total (*n*) frequencies are incorporated. The same concept applies for non-shared mutational frequencies (*F_d_*). The ratio of the summation products is then subtracted from 1 to give a Similarity Index, or a confidence of relatedness, for the outbreak strains.

**Figure 2:**
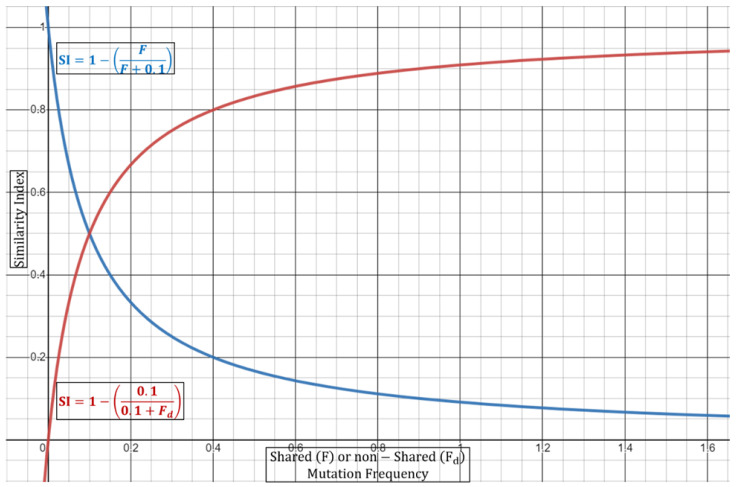
Graphical representation of modified Equation (3). Shared (*F*) or non-shared (*Fd*) combined frequencies oppositely shift the Similarity Index towards or away from 1 (100%). Blue line represents constant combined frequency of non-shared mutation with variable frequencies of shared mutations. Red line represents constant combined frequency of shared mutations with variable frequencies of non-shared mutations.

Equation (3) can be performed either in a dual fashion (two strains) or via a multi-comparison approach wherein all strains are weighted together. In the case of duality, the (*si*) as the exponent will always be equivalent to 2, since by default there are only two samples. It is important to note here that if the ORF1a regions are identical, then Equation (3) cannot be used as it depends on shared vs. non-shared ratios. In this case, the full summation expression should be equivalent to 0 to give a Similarity Index of 1 (or 100% confidence in identity). In this case, it may be of use to include other genomic regions for inferring potential mutational differences. Next, as discussed above, the Similarity Index in Equation (3) will be dependent on the increase/decrease in the number of shared or different mutations. It will also depend on the number of samples carrying a certain mutation along with the mutational frequency. Table 2 summarizes some aspects of the normalization incorporated in Equation (3) and their effects on the Similarity Index. With regards to the combined mutational frequencies, this can be observed geometrically wherein shared (*F*) or non-shared (*Fd*) combined frequencies oppositely shift the Similarity Index towards or away from 1 (100%) (Figure 2).

### 3.3. Incorporation of Spatial Dynamics

Incorporation of distance into the overall Similarity Index is useful when comparing only two patients suspected of direct contact and with confirmed interactions such as a nurse physically assisting an infected patient or simply being in the same room. One may also estimate the general average distance observed between all outbreak patients (>2) and incorporate into the Similarity Index as such. The limitation here is that some infections occur indirectly through the action of touching mucous sites with un-sanitized hands where direct patient-to-patient contact was not a factor.

The CDC’s guidelines states that the risk of CoV-2 transmission is greatest within three to six feet of an infectious source. The risk is reduced post six feet but is not eliminated due to multiple variables, such as timing compounded with other factors. Specifically, even if one is distant from the infectious source by more than six feet, the chance of transmission increases if they are in the space for longer than 15 minutes (CDC). The chance of infection increases in enclosed spaces with inadequate ventilation or if the infected person is undergoing physical and vocal exertion (e.g., exercise, singing) due to increased dispersion of virions. Nonetheless, incorporation of temporal dynamics within the Similarity Index equation will require more studies to integrate most factors with optimal mathematical presumptions precisely.

In order to incorporate patient-to-patient spatial dynamics at its simplest form, we propose utilizing a ratio of observed distance of two patients to the suggested safe distance. The physical space can be modeled as a sphere with volume, $V=\frac{4}{3}\pi r^{3}$. The geometry of the sphere (i.e., full vs. half) is not relevant since we are targeting a ratio. Therefore, the relationship can be written as such:

$$Distance\;Ratio=DR=\frac{\frac{4}{3}\pi r^{3}}{\frac{4}{3}\pi k^{3}}=\frac{r^{3}}{k^{3}}$$

where *r* is the minimum radius observed between patients; *k* is the distance constant (7 feet—in alignment with recommendations of the CDC). The distance constant should be subject to amendment depending on observational/empirical studies.

From this, the new Similarity Index with simple spatial dynamics can be written as:

(4) $$SI=1-\left(\frac{r^{3}}{k^{3}}\times \frac{\sum_{i=1}^{n}F^{si\;}i}{\sum_{i=1}^{n}F^{si\;}i+\sum_{i=1}^{n}F_{d}^{si}i}\right)$$

**Figure 3:**
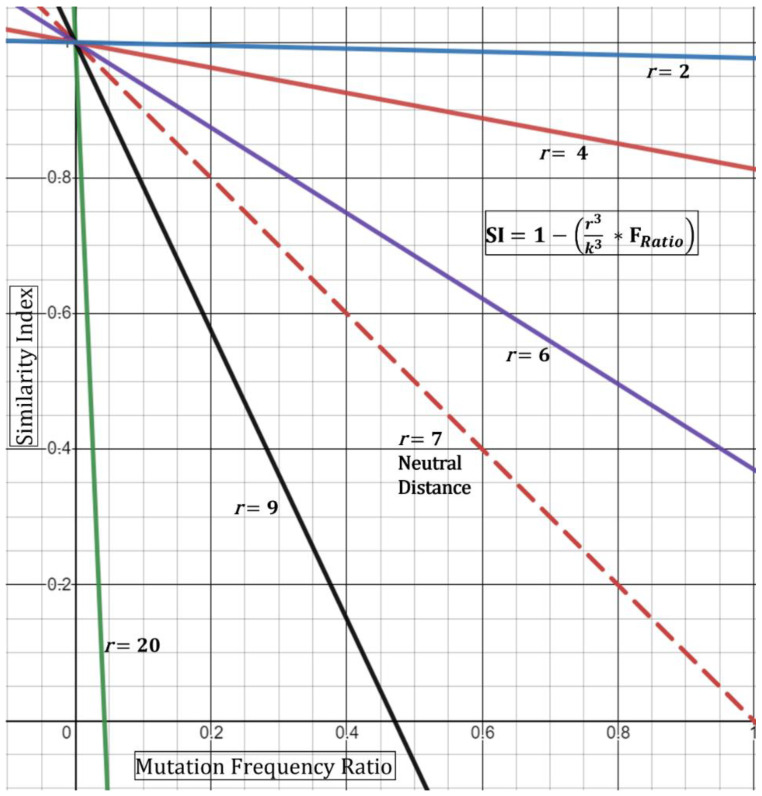
Graphical representation of modified spatial Equation (4). The observed distance/radius, *r*, of two patients behaves as a tuning knob for the Similarity Index. Close distance encounters (*r* < 7) increase the probability (SI) that the two strains are similar.

Based on this, if the minimum distance *r* observed is 7 feet, then the *DR* ratio collapses to “1” producing a “neutral” result (i.e., does not affect the index). If, however, the minimum distance is observed at 3.5 feet, then the ratio equates to 0.5, thence increasing the index—in other words, closer distances increase the chance that the strains are shared. This is geometrically displayed in (Figure 3) in its simplest form utilizing the hypothetical Essen-Möller frequency ratio (F_Ratio_). Here, as the radius *r* approaches a distance of 0 feet, the Similarity Index approaches 1 (100%), regardless of the frequency ratio. This would be true, based on the model, even if the frequency ratio of mutations observed,

$$\frac{\sum_{i=1}^{n}F^{si}i}{\sum_{i=1}^{n}F^{si}i+\sum_{i=1}^{n}F_{d}^{si}i}$$

is equivalent to >0.8 (>80%). Conceptually, the fact that two patients were observed to interact at less than two feet while one of them was a known symptomatic CoV-2 positive substantially increases the Similarity Index even if there are no unique shared mutations. We must emphasize the assumptions in Equation (4)—that is, the patients should have confirmed or have been observed to be close together. Second, patient “zero” must have been confirmed to be infected around the time of the encounter to patient “one”. Third, patient “one” would need to test positive in a short period of time and/or show symptoms of infection. The latter point is already evident for many of the outbreak investigation samples.

A limitation with the spatial Similarity Index Equation (4), as seen in (Figure 3), is that high *r* values (above 7 feet) reduce the geometric curves ever towards the y-axis and limit the highest possible normalized frequency that can be used (i.e., resultant of

$$\frac{\sum_{i=1}^{n}F^{si}i}{\sum_{i=1\;}^{n}F^{si}i+\sum_{i=1}^{n}F_{d}^{si}i\;}$$

For example, at a *r* = 9, a resultant frequency expression of ~0.47 would produce an index of 0 (Figure 3) and with any frequency above 0.47 producing a negative index. Although this demonstrates the mathematical limits of this spatial equation, conceptually it means that similarity is unlikely—with an increased negative Similarity Index correlating with higher unlikeliness. Next, we do not foresee these limits to be reached incessantly given that, with our experience, most resultants of

$$\frac{\sum_{i=1\;}^{n}F^{si}i}{\sum_{i=1}^{n}F^{si}i+\sum_{i=1\;}^{n}F_{d}^{si}i}$$

show low frequency-ratio computations. Additionally, it would be seemingly rare to receive suspect localized outbreak samples with patients who were never observed less than 20–30 feet apart.

A second limitation for the spatial Similarity Index Equation (4) is that the curves generated by the distance ratio (*DR*, and Figure 3) are linear and would not fully represent real-time kinetics. A true representation of real-time spatial effects must include a temporal component where both factors are then guided by the physics of viral transmission, infectivity (Ct value), and length of exposure [12]. Case-A analysis below demonstrates the use of Equation (4) where two of five specimens that are qualitatively expected to be similar had a reduced Similarity Index. Nonetheless, the equation should be used with full understanding of these limitations.

### 3.4. Analysis of Outbreak Samples via Similarity Index–Proof of Concept

To test our model, we utilized two cases (case-A and case-B) from our clinical reports on which we have concluded qualitatively as an outbreak case with similar and dissimilar strains, respectively. With regard to case-A, although it was qualitatively deemed to contain shared strains, there were samples that were more similar to each other when comparing nucleotide sequences within ORF1a. We utilized Equation (4) with a DR of one (i.e., neutral spatial dynamics). Table 3 shows the mutations found within CoV-2 strains (extracted from suspected outbreak patients), while Table 4 describes the Similarity Index between each pair of strains and the group. As expected, we can see that most samples have an index of >98%. The most dissimilarity was observed between p-1 and p-4 along with p-2 and p-4 showing a Similarity Index of 62.83% (Table 4). This is logical as patients 1 and 2 are identical at ORF1a (Table 4).

Interestingly, we observe here that p-1 and p-3 have a higher Similarity Index than p-1 and p-4. This seemed paradoxical since p-1 and p-4 have an extra shared mutation compared to p-1 and p-3 (Table 4). The reason is that this extra shared mutation appears at a high frequency (68%). Albeit this seems paradoxical, it demonstrates that Equation (4) reduced the certainty of strain similarity since approximately 68% of circulating CoV-2 strains share this mutation and that the equation is based on random selection. It simply tells us that we have reduced confidence that p-1 and p-4 are similar given that they are chosen at random. This is one reason why it becomes important to include spatiotemporal components. These components integrate the important evidence, that the patients were close together in space and time during a known period of infection thence reducing the element of randomness with the Similarity Index. To this end, we shall hypothetically assume that p-1 and p-4 were observed at 5 feet, while one was symptomatic with a known positive low qRT-PCR Ct at the time of observation. In this case, using Equation (4) with a spatial component of 5 feet, the index is increased from 62.828% to 86.453%. Overall, these results show that lower frequency mutations are highly important in the differentiation process (i.e., excluding dissimilarity).

Using Equation (4), the combined Similarity Index was at 99.999%, but we also attempted to average the single indices for all five patients, producing an average of 92.135%. The discrepancy between Equation (4)-combined and the averaged single indices can be explained by the differential inclusion of all mutations with the total number of samples carrying shared vs. non-shared mutations, as detailed in the derivations section. Here, the combined Similarity Index by Equation (4) is heavily affected by low frequency mutations being shared, which increases the confidence that all samples are derived from the same source. In this case, the non-shared mutations had higher frequencies than the shared, which as we expect swayed the calculated values towards confidence of similarity (Table 4).

Next, we analyzed case-B which we qualitatively reported clinically as a dissimilar group. We can observe that, although these three patients share plenty of mutations, they mostly nevertheless appear at high frequencies (Table 5). Additionally, all three patients harbor non-shared low frequency mutations. As expected, the Similarity Index for all patients was overall lower than 5% (Table 6). Thus, the equation demonstrates that even if all strains in question share several high frequency mutations, it still may not provide enough confidence to define similarity. Instead, high frequency mutations drive the index towards lower values due to reduced confidence stemming from the presence of high-prevalence mutations. The latter, occurring in the presence of non-shared low frequency mutations, substantially reduces the confidence in strain sharing as seen in this case. To this end, the combined Similarity Index by Equation (4) is calculated at 6.429% with averaged single indices at 3.031%.

## 4. Conclusions

We herein proposed the use of a theoretical probability-based model for defining relatedness between CoV-2 viral strains in localized outbreak scenarios. We showed the utility of this equation with two outbreak evaluations performed at our center. The proposed inputs include outbreak sample number, mutational frequencies, and spatial dynamics, thence producing a probability or a likelihood of association (i.e., Similarity Index). The utility of this value was demonstrated utilizing outbreak cases wherein the interplay of the aforementioned factors controlled the outcome of relatedness. Nevertheless, we list potential limitations, within the above arguments, associated with the use of input variables. We propose that there is a need for empirical evidence in order to provide more accurate constants for the Similarity Index. The latter includes more defined spatial parameters along with temporal dynamics and inclusion of infectivity factors (i.e., qRT-PCR Ct values).

It is important to note here that the index, produced by Equations (3) and (4), is reflecting our confidence in strain relatedness when chosen from a random population of infected individuals, and not necessarily how related the strains are in absolute terms (genetically). An example would be when the strains of interest are almost identical but only share common high frequency mutations. In this case, even though the strains appear near identical in the mutational repertoire, we are nevertheless not confident that they are from the same spatiotemporal source (related) since most circulating CoV-2 samples would be sharing these mutations. This is where additional criteria can be added including evidence of spatial connection between two samples which will then increase the Similarity Index. On the other hand, if all strains are 100% identical in the mutational repertoire, even with common mutations, then this would give an index of 100% confidence that they are same especially when we have the evidence of physical transmission in the same period.

One concern that may arise from this argument is the single use of one genomic region (ORF1a). There are two reasons behind the use of one gene, specifically ORF1a. First, when a certain clade is dominant (as was observed with Delta), the classically varied regions between clades, such as the Spike gene, are in fact relatively stable within a single clade. It then becomes difficult to compare localized outbreak samples when the Spike region is notably stable and homologues. In contrast, the ORF1a gene is relatively unstable (i.e., informative). We observed, at least within Delta, that ORF1a mutations vacillate and drift spatiotemporally thereby allowing the potential for comparisons. We suppose that if potentially upcoming variants show stability (i.e., non-informative regions) within ORF1a, then other instable genes can instead be considered. We have thus far observed that Omicron virions behave in a similar fashion to Delta, within the ORF1a gene, whereby enough instability occurs for proper comparisons of outbreak strains. We emphasize here that frequency values utilized for Omicron, or other emerging strains, should utilize the appropriate current timeframe to exclude mutational data for non-circulating strains such as the Delta variant.

Second, genetically (by nucleotide basis) evaluating the full genome between outbreak strains can be misleading. The reason is that full genome comparisons of the same species are too general and are rather more beneficial when comparing different species or genus (i.e., CoV-2 to CoV-1, or CoV-2 to HCoV-HKU1). An example of this is demonstrated with forensic science whereby genomic DNA from a victim and a suspect are compared. In this case, specific polymorphic (i.e., instable/informative) regions such as Short Terminal Repeats (STR) are utilized to define similarities/differences and attain a probability to use for/against the suspect [7]. We do not compare whole genomes which will misleadingly produce a false-positive result of high similarity since most of the nucleotide sequences (between two humans) are generally identical. These types of whole genome comparisons are more practical when comparing human vs. other species’ DNA. In essence, this is where polymorphic regions become valuable in forensics, such as STR. Thus, constricting the genomic analysis in a viral strain to one unstable genomic region provides a more accurate extrapolation in terms of strain relatedness. Therefore, this method presented here should not be used to compare viruses from different species, as the assumption is that one species (which is genetically homologous) is being compared. Overall, this model can function as a platform for future refinements and developments including implementation in the analysis of localized outbreaks with other pathogens.

## Figures and Tables

**Table 1:** P of non-random Association (Frequency vs. Sample Number).

Frequency	*p* (2 Samples)	*p* (3 Samples)	*p* (5 Samples)
0.6	0.6400	0.7840	0.9222
0.5	0.7500	0.8750	0.9688
0.3	0.9100	0.9730	0.9976
0.15	0.9775	0.9966	0.99992406
0.1	0.9900	0.9990	0.99999
0.05	0.9975	0.9999	0.99999968

**Table 2:** Effects on Similarity Index Outcome (Equation (3)) ↑/↓.

Parameter	Increase	Decrease
Samples Sharing a Mutation	↑	↓
Samples with a Differing Mutation	↓	↑
Absolute Frequency of a Shared Mutation (*F*)	↓	↑
Absolute Frequency of a Differing Mutation (*Fd*)	↑	↓

**Table 3:** Case-A ORF1a Mutations and Frequencies.

Mutation	C884 T	C1191 T	C3737 T	C5184 T	C6402 T	G9203 A	T9678 C	C11005 A	A11201 G
Frequency	0.004188	0.035824302	0.001129679	0.109622311	0.683634019	0.0095136	0.009166893	0.009759221	0.685742635
P1		X	X		X	X	X	X	X
P2		X	X		X	X	X	X	X
P3	X	X	X	X		X	X	X	
P4	X	X	X	X		X	X	X	X
P5	X	X	X	X		X	X	X	

Mutations observed within ORF1a gene between all outbreak samples. “X” denotes the presence of the mutation. The frequency of each mutation is listed below its designation. P1–P5 imply CoV-2 extracted from outbreak patient-1 to patient-5.

**Table 4:** Case-A Similarity Index (SI). Case-A calculated SI utilizing Equation (4).

	P1	P2	P3	P4	P5	Combined Similarity Index
P1		100.000	99.895	62.828	99.895	99.999 (Averaged Single SI = 92.135)
P2			99.895	62.828	99.895	
P3				98.057	100.000	
P4					98.057	
P5						

**Table 5:** Case-B ORF1a Mutations and Frequencies. Mutations observed within ORF1a gene between all outbreak samples. “X” denotes the presence of the mutation. The frequency of each mutation is listed below its designation. P1–P3 imply CoV-2 extracted from outbreak patient-1 to patient-3.

Mutation	C1191 T	G4181 T	C6402 T	C7124 T	G9053 T	C10029 T	A10323 G	A11201 G	A11456 G
Frequency	0.0358243	0.68507671	0.683634019	0.66987836	0.684267136	0.697208788	0.014608	0.685742635	0.081708422
P1		X	X	X	X	X	X	X	
P2	X	X	X	X	X	X		X	
P3		X	X	X	X	X		X	X

**Table 6:** Case-B Similarity Index (SI). Case-B calculated SI utilizing Equation (4).

	P1	P2	P3	Combined Similarity Index
P1		1.763	3.314	6.429 (Averaged Single SI = 3.031)
P2			4.015	
P3				

## Data Availability

The data presented in this study are available upon request from the corresponding author.
